# Comparative Analysis of Outcomes of Influenza and COVID-19 Admissions Among Children With Asthma: A Nationwide Retrospective Cohort Study Using the US National Readmissions Database

**DOI:** 10.2196/73047

**Published:** 2025-09-30

**Authors:** Ying-Chen Chen, Chia-Pi Cheng, Po-Cheng Chen, Jinn-Li Wang, Chia-Chen Wu, Ying-Chun Lu

**Affiliations:** 1 Department of Pediatrics Tri-Service General Hospital, National Defense Medical University Taipei Taiwan; 2 Division of Rheumatology/Immunology/Allergy, Department of Internal Medicine Tri-Service General Hospital, National Defense Medical University Taipei Taiwan; 3 Department and Graduate institute of Biology and Anatomy, National Defense Medical Center Taipei Taiwan; 4 Division of Pediatric Rehabilitation, Department of Physical Medicine and Rehabilitation, Wo On Rehabilitation Clinic New Taipei Taiwan; 5 Division of Hematology and Oncology, Department of Pediatrics, Wan Fang Hospital, Taipei Medical University Taipei Taiwan; 6 Department of Pediatrics, School of Medicine, College of Medicine Taipei Medical University Taipei Taiwan; 7 Department of Surgery, Tri-Service General Hospital Keelung Branch National Defense Medical Center Keelung Taiwan; 8 Division of Thoracic Surgery, Department of Surgery, Tri-Service General Hospital National Defense Medical Center Taipei Taiwan; 9 Division of Neonatology, Department of Pediatrics Wan Fang Hospital, Taipei Medical University Taipei Taiwan; 10 Division of Pediatric Critical Care Medicine, Department of Pediatrics, Wan Fang Hospital Taipei Medical University Taipei Taiwan; 11 Division of Pediatric Emergency Medicine, Wan Fang Hospital Taipei Medical University Taipei Taiwan

**Keywords:** asthma, children, influenza, COVID-19, National Readmission Database, NRD

## Abstract

**Background:**

Asthma is a common chronic respiratory disease with increasing prevalence among children over the past few decades. It can cause significant respiratory symptoms and acute exacerbations, often requiring emergency care or hospitalization. Moreover, exposure to respiratory viral infections, such as COVID-19 and influenza, can trigger severe complications in children with asthma. Despite these concerns, few studies have directly compared the in-hospital outcomes of children with asthma experiencing these infections.

**Objective:**

This study aimed to compare the in-hospital outcomes of these infections in children with asthma from a population-based perspective.

**Methods:**

We conducted a population-based retrospective cohort study using data from the 2020 US Nationwide Readmissions Database. Children aged 1 to 19 years with asthma who were admitted for COVID-19 or influenza were eligible for inclusion. Outcomes evaluated included in-hospital mortality, major complications, and 90-day readmission rate. Survey-weighted logistic regression models were used to compare clinical outcomes between the two infection groups, adjusting for demographic and clinical characteristics.

**Results:**

A total of 1472 hospitalized children with asthma were included, of whom 405 (27.5%) were admitted for COVID-19 and 1067 (72.5%) for influenza. After adjustment, the multivariate analysis revealed that children admitted for COVID-19 had a significantly higher risk of sepsis or shock (adjusted odds ratio [aOR] 4.30, 95% CI 1.79-10.32) but a lower risk of bacterial or fungal pneumonia (aOR 0.37, 95% CI 0.23-0.61) compared with those admitted for influenza. Stratified analyses by age revealed that among children aged 1 to 5 years, the risk of 90-day readmission was significantly higher for those with COVID-19 than for those with influenza (aOR 3.02, 95% CI 1.09-8.35). No significant difference in in-hospital mortality was detected between the two infection groups in either the multivariable model or any of the age-stratified analyses.

**Conclusions:**

US children with asthma hospitalized for COVID-19 had higher risks of sepsis or shock compared to those admitted for influenza. In contrast, children admitted for influenza had a higher risk for bacterial or fungal pneumonia. After stratifying by age, children aged 1 to 5 years with COVID-19 had a significantly higher risk of 90-day readmission than those with influenza. Our findings suggest that different clinical approaches may be needed for children with asthma, depending on infection etiology and patient age.

## Introduction

### Background

Asthma prevalence has risen steadily worldwide over the past 4 decades [1]. The incidence of childhood asthma has markedly increased over the past 40 years. Despite therapeutic advances, underdiagnosis and undertreatment remain common in children with asthma [2,3].

Respiratory viral infections such as COVID-19 and influenza often precipitate severe asthma exacerbations in children. Recent research indicates that asthma increases the risk of hospitalization in children with COVID-19 [4,5]. In addition, individuals with uncontrolled asthma are more likely to experience severe illness from COVID-19 that requires hospitalization [6]. Similarly, influenza can also lead to a worsening of asthma symptoms and may require hospitalization [7].

Although children with asthma who contract COVID-19 or influenza often experience more severe disease than their peers without asthma, there is a lack of studies examining the in-hospital outcomes of children with asthma who develop COVID-19 or influenza. Lee et al [8] analyzed the Korean Childhood Asthma Study cohort and showed that health care use among children with asthma significantly decreased during the COVID-19 pandemic, suggesting that only more severe cases reached hospital care. Furthermore, longitudinal work by Yoon et al [9] identified distinct phenotypic trajectories of childhood asthma with allergic comorbidities, underscoring the importance of age-specific risk estimates. However, no national study has directly compared the in-hospital outcomes of children with asthma admitted with COVID-19 versus those admitted with seasonal influenza.

### Objectives

The purpose of this study was to compare in-hospital mortality, major complication rates, and 90-day readmission rates between children with asthma admitted for COVID-19 and those admitted for influenza using a national US database.

## Methods

### Data Source

The Nationwide Readmissions Database (NRD) is a database of all-payer hospital inpatient stays that can be used to generate national estimates of readmissions [10]. The NRD is a 100% sample from the Health Care Cost and Utilization Project (HCUP) State Inpatient Databases with discharge-level and hospital-level exclusions. The database contains verified patient linkage numbers that can be used to track a person across hospitals within a state while adhering to strict privacy guidelines. Researchers can identify diagnoses and procedures in the NRD using the *ICD-10-CM* (*International Classification of Diseases, 10th Revision, Clinical Modification*) and the *ICD-10-PCS* (*International Classification of Diseases, 10th Revision, Procedure Coding System*) codes. We used deidentified patient records from hospital discharges across multiple states, which allowed researchers to track readmissions nationally. Our research team obtained access to the NRD through the HCUP central distributor as per a data use agreement. The database is not open access in the sense of being freely downloadable; however, it is accessible to researchers worldwide who complete the requisite training and agree to the terms of the HCUP.

### Study Design and Population

This population-based retrospective cohort study used NRD data from the year 2020. Inclusion criteria for this study were (1) children aged 1 to 19 years; (2) first admission for COVID-19 or influenza between January 1 and September 30, 2020, identified by the relevant *ICD-10-CM* codes documented as the principal diagnosis; and (3) asthma as a secondary diagnosis. Children with missing information on the main end points, sex, and sample weights were excluded. Following the index admission, patients were considered *at risk* for hospitalization and were followed until December 31 of the admission year or until death. Detailed *ICD-10-CM* codes used are provided in Multimedia Appendix 1.

### Ethical Considerations

This study is a secondary analysis and adheres to the terms of the HCUP data use agreement. Specifically, the data were acquired from the online HCUP central distributor and were anonymized and publicly available. According to 45 CFR §46.104(d)(4) of the US Common Rule, secondary research on publicly available, fully deidentified data is exempt from institutional review board (IRB) review [11]. Hence, this study was exempt from IRB approval as per our institutional guidelines. Therefore, no informed consent or further IRB approval was required.

### Variables and Outcome Measures

Data collected included patient age, sex, insurance status, and household income. Asthma was defined exclusively by *ICD-10-CM* code J45, which is assigned by trained coders only when a treating clinician documents a definite diagnosis of asthma. Furthermore, data relating to hospital characteristics such as weekend admission, admission type (emergent or elective), hospital bed size, and location or teaching status were collected. Major comorbidities extracted from the medical records and included in the analysis were hypertension, diabetes mellitus, obesity or overweight, neurological diseases, Down syndrome or chromosomal anomalies, metabolic diseases, cystic fibrosis, sickle cell disease, congenital heart conditions, congenital lung conditions, autoimmune disease, and mental or physical disabilities. These are comorbidities frequently considered in hospital admissions of children.

The outcomes evaluated included in-hospital mortality and complications, including sepsis or shock, secondary bacterial or fungal pneumonia, respiratory failure or mechanical ventilation, and acute kidney injury. In addition, 90-day readmission rates were assessed.

### Statistical Analysis

The NRD includes discharge-level weights, stratum variables, and cluster Hospital identifier variable in the Nationwide Readmissions Database variables, which were used to estimate discharges from community hospitals in the United States, excluding rehabilitation and long-term acute care facilities. These variables were also used to calculate SEs and produce national estimates for all analyses in this study. In SAS statistical software (SAS Institute), the survey procedure is available for the analysis of sample survey data. Descriptive statistics of patients at the time of index admission were presented as either number and weighted percentage or mean and SE, and patients were categorized into the COVID-19 or influenza group. Categorical data were examined using the PROC SURVEYFREQ (survey frequency procedure) statement, while continuous data were assessed using the PROC SURVEYREG (procedure for survey regression analysis) statement. The SURVEYFREQ procedure includes the Rao-Scott chi-square test to assess the significance between weighted proportions. The SURVEYREG procedure fits linear models for survey data and provides significance tests for the model effects. For univariate analyses, no correction was applied for multiple comparisons, as these were considered exploratory. This approach is supported by methodological literature, which suggests that adjustment for multiplicity is not always necessary in such contexts [12].

Logistic regressions were performed using the PROC SURVEYLOGISTIC (procedure for survey logistic regression analysis) statement to compare the risk of adverse outcomes between children admitted for COVID-19 or influenza, including in-hospital mortality, major complications, and 90-day readmission, adjusting for covariates with significance in the univariate analysis in a multivariable model. Data were reported as odds ratios and 95% CI. All *P* values were 2-sided, and the value of *P*<.05 was considered statistically significant. Statistical analyses were performed using SAS software (version 9.4; SAS Institute).

## Results

### Patient Selection

**Figure 1:**
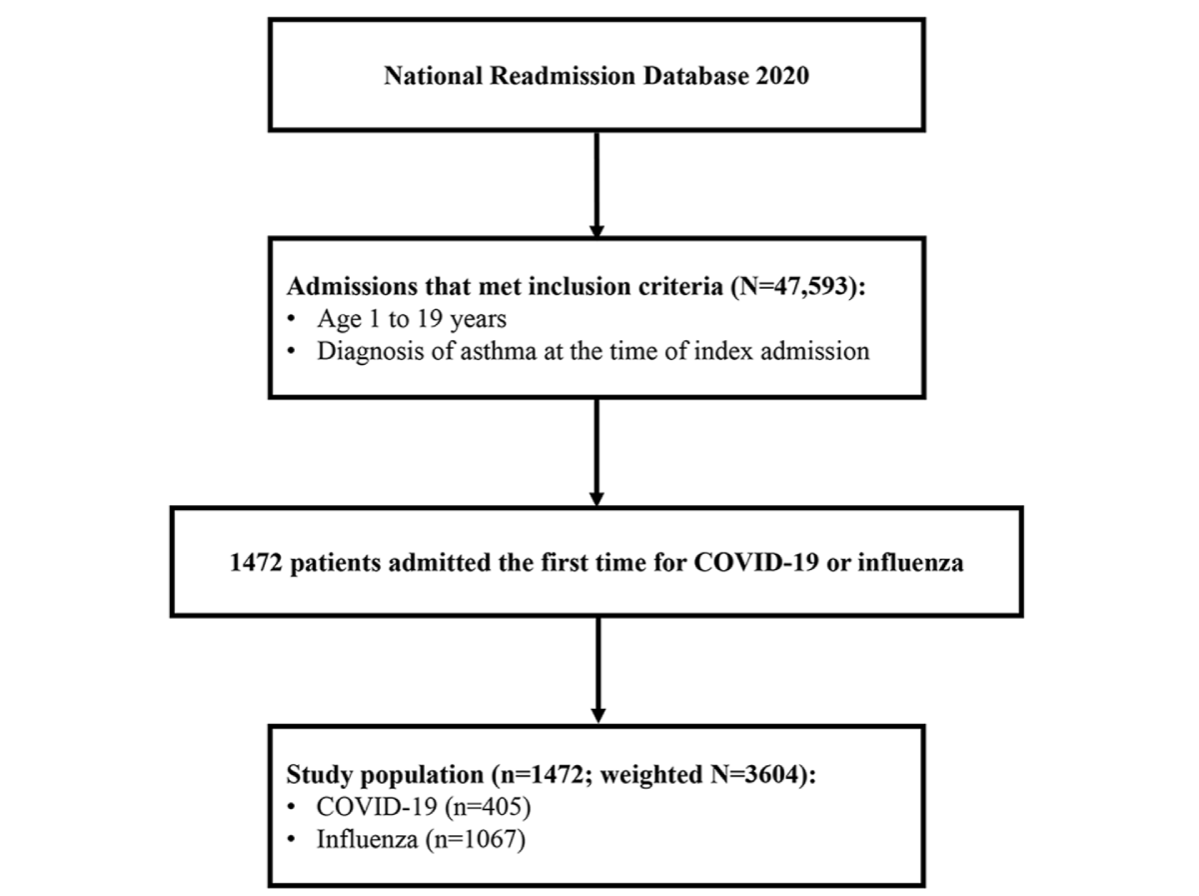
Flow diagram of patient selection for a nationwide retrospective cohort study of US children aged 1–19 years with asthma who were hospitalized for a first diagnosis of COVID-19 or seasonal influenza between Jan 1 and Dec 31, 2020, in the Nationwide Readmissions Database (NRD); final analytic sample: n=1472 (weighted N=3604).

The selection process for the study population is illustrated in Figure 1. A total of 1472 patients aged 1 to 19 years with asthma who were admitted for the first time with COVID-19 or influenza were identified in the 2020 NRD. Of the 1472 patients, 405 (27.5%) were admitted for COVID-19 and 1067 (72.5%) for influenza. This sample represented 3604 individuals who were hospitalized in the entire United States after applying the sample weights provided by the NRD.

### Patient Characteristics

Patient characteristics are summarized in Table 1. The mean age of all patients was 8.5 (SD 0.2 years), and 59% (862/1472) were male individuals. Significant differences were observed in age, household income, insurance status or primary payer, hospital bed size, and location or teaching status between patients with COVID-19 and those with influenza. In addition, the proportions of patients with diabetes mellitus, hypertension, obesity or overweight, metabolic diseases, sickle cell disease, and congenital heart conditions were significantly different between patients with COVID-19 and those with influenza.

**Table 1:** Demographic, socioeconomic, hospital, and comorbidity characteristics of US children (aged 1-19 years) with physician-diagnosed asthma admitted for COVID-19 (n=405) or seasonal influenza (n=1067) in the 2020 Nationwide Readmissions Database.

Characteristics			Total (N=1472)	COVID-19 (n=405)	Influenza (n=1067)	*P* value	
**Adverse outcomes, n (%)**							
	In-hospital mortality		13 (0.9)	10 (2.5)	3 (0.4)	*.003* ^a^	
	**Major complications, any**		313 (22.1)	94 (22.7)	219 (22)	.80	
		Sepsis or shock	47 (2.9)	35 (8.4)	12 (1)	*<.001*	
		Secondary bacterial or fungal pneumonia	202 (15.1)	28 (7.6)	174 (17.7)	*<.001*	
		Respiratory failure or mechanical ventilation	84 (5.6)	40 (9.1)	44 (4.3)	*<.001*	
		AKI^b^	52 (4)	34 (9)	18 (2.2)	*<.001*	
	90-day readmission rate^c^		84 (6)	27 (6.9)	57 (5.7)	.38	
**Demography**							
	**Age (y), mean (SE)**		8.5 (0.2)	13.3 (0.3)	6.8 (0.2)	*<.001*	
		1 to 5, n (%)	557 (37.9)	50 (12.5)	507 (46.9)		
		6 to 10, n (%)	381 (27.1)	51 (14.1)	330 (31.7)		
		11 to 15, n (%)	234 (17)	95 (26)	139 (13.8)		
		16 to 19, n (%)	300 (18)	209 (47.4)	91 (7.6)		
	**Sex, n (%)**						.27
		Male	862 (59.2)	227 (56.8)	635 (60)		
		Female	610 (40.8)	178 (43.2)	432 (40)		
	**Household income, n (%)**						*.02*
		Quartile 1	488 (32.2)	159 (38.7)	329 (29.9)		
		Quartile 2	393 (27.2)	107 (27)	286 (27.3)		
		Quartile 3	338 (24)	85 (21.3)	253 (24.9)		
		Quartile 4	242 (16.6)	48 (13)	194 (17.9)		
		Missing	11	6	5		
	**Insurance status or primary payer, n (%)**						*<.001*
		Medicare or Medicaid	886 (58.8)	281 (68.6)	605 (55.3)		
		Private including HMO^d^	518 (36.8)	105 (26.7)	413 (40.4)		
		Self-pay, no charge, or other	68 (4.4)	19 (4.7)	49 (4.3)		
	**Admission type, n (%)**						.58
		Elective	40 (2.8)	8 (2.4)	32 (2.9)		
		Emergent	1431 (97.2)	397 (97.6)	1034 (97.1)		
		Missing	1	0	1		
**Hospital-related characteristics, n (%)**							
	**Hospital bed number**						*.03*
		Small	179 (13.2)	34 (8.8)	145 (14.7)		
		Medium	306 (21.8)	71 (19.3)	235 (22.6)		
		Large	987 (65.1)	300 (71.9)	687 (62.7)		
	**Location or teaching status**						*.02*
		Metropolitan nonteaching	99 (7.3)	21 (4.8)	78 (8.2)		
		Metropolitan teaching	1304 (88.8)	372 (92.6)	932 (87.4)		
		Nonmetropolitan	69 (3.9)	12 (2.6)	57 (4.4)		
	**Weekend admission**						.41
		No	1084 (73.8)	291 (72.1)	793 (74.4)		
		Yes	388 (26.2)	114 (27.9)	274 (25.6)		
**Comorbidities**							
	DM^e^		41 (2.5)	32 (6.8)	9 (1)	*<.001*	
	Hypertension		44 (2.7)	31 (7.1)	13 (1.2)	*<.001*	
	Obesity or overweight		157 (9.7)	122 (28.1)	35 (3.2)	*<.001*	
	Neurological diseases		138 (9.4)	44 (12)	94 (8.5)	.05	
	Down syndrome or chromosomal anomalies		33 (2.3)	9 (2.3)	24 (2.3)	.99	
	Metabolic diseases		55 (3.9)	32 (8.6)	23 (2.3)	*<.001*	
	Cystic fibrosis		9 (0.7)	3 (0.7)	6 (0.7)	.985	
	Sickle cell disease		75 (5.4)	34 (8.5)	41 (4.3)	*.003*	
	Congenital heart conditions		21 (1.4)	14 (3.3)	7 (0.7)	*<.001*	
	Autoimmune diseases		31 (2.1)	15 (3.2)	16 (1.7)	.08	
	Disabilities		73 (4.9)	20 (4.6)	53 (5)	.73	

^a^*P* values <.05 are italicized.

^b^AKI: acute kidney injury.

^c^Data excluding patients who died in the hospital.

^d^HMO: Health Maintenance Organization.

^e^DM: diabetes mellitus.

Patients admitted for COVID-19 had higher frequencies of in-hospital mortality (2.5% vs 0.4%; *P*=.003), sepsis or shock (8.4% vs 1%; *P*<.001), respiratory failure or mechanical ventilation (9.1% vs 4.3%; *P*<.001), and acute kidney injury (9% vs 2.2%; *P*<.001) compared to those admitted for influenza. On the other hand, the proportion of patients with secondary bacterial or fungal pneumonia was significantly higher in the group admitted for influenza compared to the group admitted for COVID-19 (18% vs 8%; *P*<.001).

### Comparison of Adverse Outcomes Between Children Admitted for COVID-19 and Those Admitted for Influenza

A comparison of outcomes between children admitted for COVID-19 and influenza is provided in Table 2. After adjustment in the multivariable analysis, patients admitted for COVID-19 had a significantly higher risk of sepsis or shock (adjusted odds ratio [aOR] 4.30, 95% CI 1.79-10.32; *P*=.001) but a lower risk of secondary bacterial or fungal pneumonia (aOR 0.37, 95% CI 0.23-0.61; *P*<.001) compared to those admitted for influenza (Table 2).

**Table 2:** Unadjusted and adjusted odds ratios (aORs) for in-hospital mortality, major complications, and 90-day readmission among US children with asthma (aged 1 to 19 years) admitted for COVID-19 versus seasonal influenza, Nationwide Readmissions Database, calendar year 2020.

Adverse outcomes		Univariate			Multivariable	
		OR^a^ (95% CI)	*P* value	Adjusted OR (95% CI)		*P* value
In-hospital mortality^b^		6.15 (1.51-24.99)	*.01* ^c^	2.18 (0.56-8.49)		.26
**Major complications^d^**		1.04 (0.76-1.42)	.79	0.90 (0.67-1.21)		.50
	Sepsis or shock^e^	9.23 (4.18-20.36)	*<.001*	4.30 (1.79-10.32)		*.001*
	Secondary bacterial or fungal pneumonia^f^	0.38 (0.23-0.64)	*<.001*	0.37 (0.23-0.61)		*<.001*
	Respiratory failure or mechanical ventilation^g^	2.24 (1.39-3.61)	*<.001*	1.47 (0.83-2.61)		.18
	Acute kidney injury^h^	4.45 (2.24-8.85)	*<.001*	1.97 (0.92-4.22)		.08
90-day readmission rate^i,j^		1.23 (0.77-1.96)	.39	0.95 (0.56-1.62)		.85

^a^OR: odds ratio.

^b^Adjusted for all variables significant in the univariate analysis: age (continuous), diabetes mellitus, neurological diseases, metabolic diseases, and disability.

^c^*P* values <.05 are italicized.

^d^Adjusted for all variables significant in the univariate analysis: insurance status or primary payer, diabetes mellitus, neurological diseases, metabolic diseases, sickle cell disease, and congenital heart conditions.

^e^Adjusted for all variables significant in the univariate analysis: age (continuous), diabetes mellitus, obesity or overweight, neurological diseases, metabolic diseases, congenital heart conditions, and disability.

^f^Adjusted for all variables significant in the univariate analysis: insurance status or primary payer, neurological diseases, and sickle cell disease.

^g^Adjusted for all variables significant in the univariate analysis: age (continuous), household income, diabetes mellitus, hypertension, obesity or overweight, neurological diseases, metabolic diseases, congenital heart condition, and disabilities.

^h^Adjusted for variables significant in the univariate analysis: diabetes mellitus, obesity or overweight, neurological diseases, Down syndrome or chromosomal anomalies, metabolic diseases, and congenital heart condition.

^i^Adjusted for all variables significant in the univariate analysis: household income, hypertension, neurological diseases, metabolic diseases, cystic fibrosis, sickle cell disease, and disabilities.

^j^Excluding patients who died in the hospital.

### Comparison of Adverse Outcomes Between Children Admitted for COVID-19 and Those Admitted for Influenza, Stratified by Age

The results of the stratified analysis are shown in Table 3. Among patients aged 1 to 5 years, those admitted for COVID-19 had a significantly higher 90-day readmission rate (aOR 3.02, 95% CI 1.09-8.35; *P*=.03) and a significantly lower risk of secondary bacterial or fungal pneumonia (aOR 0.13, 95% CI 0.02-0.96; *P*=.046) compared to those admitted for influenza. In those aged 6 to 10 years, children with asthma who were admitted for COVID-19 had a significantly higher risk of sepsis or shock (aOR 6.64, 95% CI 1.23-35.91; *P*=.03). Among adolescents aged 11 to 19 years, patients admitted for COVID-19 had a significantly higher risk of sepsis or shock (aOR 4.79, 95% CI 1.79-12.84; *P*=.002) but a significantly lower risk of secondary bacterial or fungal pneumonia (aOR 0.42, 95% CI 0.24-0.73; *P*=.002; Table 3).

**Table 3:** Age-stratified adjusted odds ratios (aORs) for adverse outcomes among US children with asthma hospitalized for COVID-19 compared with seasonal influenza, Nationwide Readmissions Database, 2020.

Adverse outcomes				aOR (95% CI)		*P* value
**Age 1 to 5 years**						
	In-hospital mortality^a^		N/A^b^		—^c^	
	**Major complications^d^**		0.56 (0.13-2.34)		.43	
		Sepsis or shock^e^	1.41 (0.14-14.14)		.77	
		Secondary bacterial or fungal pneumonia^f^	0.13 (0.02-0.96)		*.046* ^g^	
		Respiratory failure or mechanical ventilation^h^	1.41 (0.23-8.77)		.71	
		Acute kidney injury^i^	3.55 (0.29-43.66)		.32	
	90-day readmission rate^j,k^		3.02 (1.09-8.35)		*.03*	
**Age 6 to 10 years**						
	In-hospital mortality^a^		2.53 (0.27-23.46)		.41	
	**Major complications^d^**		0.91 (0.39-2.10)		.82	
		Sepsis or shock^e^	6.64 (1.23-35.91)		*.03*	
		Secondary bacterial or fungal pneumonia^f^	0.42 (0.13-1.39)		.16	
		Respiratory failure or mechanical ventilation^h^	1.32 (0.45-3.88)		.61	
		Acute kidney injury^i^	2.11 (0.31-14.25)		.44	
	90-day readmission rate^j,k^		0.49 (0.13-1.81)		.28	
**Age 11 to 19 years**						
	In-hospital mortality^a^		6.02 (0.87-41.75)		.07	
	**Major complications^d^**		0.90 (0.58-1.38)		.62	
		Sepsis or shock^e^	4.79 (1.79-12.84)		*.002*	
		Secondary bacterial or fungal pneumonia^f^	0.42 (0.24-0.73)		*.002*	
		Respiratory failure or mechanical ventilation^h^	1.82 (0.61-5.42)		.28	
		Acute kidney injury^i^	1.56 (0.78-3.13)		.21	
	90-day readmission rate^j,k^		0.80 (0.37-1.74)		.57	

^a^Adjusted for all variables significant in the univariate analysis (except for stratified or N/A variables): diabetes mellitus, neurological diseases, metabolic diseases, and disabilities.

^b^N/A: not applicable. No event occurred in one group.

^c^Data not applicable.

^d^Adjusted for all variables significant in the univariate analysis (except for stratified or N/A variables): insurance status or primary payer, diabetes mellitus, neurological diseases, metabolic diseases, sickle cell disease, and congenital heart conditions.

^e^Adjusted for all variables significant in the univariate analysis (except for stratified or N/A variables): diabetes mellitus, obesity or overweight, neurological diseases, metabolic diseases, congenital heart conditions, and disabilities.

^f^Adjusted for all variables significant in the univariate analysis (except for stratified or N/A variables): insurance status or primary payer, neurological diseases, and sickle cell disease.

^g^*P* values <.05 are italicized.

^h^Adjusted for all variables significant in the univariate analysis (except for stratified or N/A variables): household income, diabetes mellitus, hypertension, obesity or overweight, neurological diseases, metabolic diseases, congenital heart conditions, and disabilities.

^i^Adjusted for all variables significant in the univariate analysis (except for stratified or N/A variables): diabetes mellitus, obesity or overweight, neurological diseases, Down syndrome or chromosomal anomalies, metabolic diseases, and congenital heart conditions.

^j^Adjusted for all variables significant in the univariate analysis (except for stratified or N/A variables): household income, hypertension, neurological diseases, metabolic diseases, cystic fibrosis, sickle cell disease, and disabilities.

^k^Excluding patients who died in the hospital.

## Discussion

### Principal Findings

In this study, we compared the outcomes of children with asthma who were hospitalized with COVID-19 or influenza using the US NRD. Our analysis showed that children with asthma hospitalized for COVID-19 had a higher risk of sepsis or shock, whereas those hospitalized for influenza had a higher risk of secondary bacterial or fungal pneumonia. There was no significant difference in in-hospital mortality between the two groups. Furthermore, age-stratified analysis indicated that children aged 1 to 5 years with COVID-19 had a 3-fold higher risk of 90-day readmission compared to those with influenza. In adolescents (aged 11 to 19 years), the COVID-19 group faced a higher sepsis or shock risk but a lower risk of secondary bacterial or fungal pneumonia. These findings highlight the different clinical challenges in managing influenza and COVID-19 in children with asthma.

### Comparison With Prior Work

Both influenza and COVID-19 are serious infections, and comorbidities such as asthma can worsen their clinical outcomes. While there have been studies comparing the outcomes of these infections in adults, there is limited research on children, especially those with asthma. Xie et al [13] found that hospitalization for COVID-19 was associated with a higher risk of death compared to that for influenza (6% for COVID-19 vs 3.7% for influenza; hazard ratio 1.16). The risk of death decreased with “with increasing numbers of COVID-19 vaccinations (*P*=.009). An earlier study comparing patients diagnosed with influenza during the 2017 to 2018 season to those diagnosed with COVID-19 between February and June 2020 found that, despite being younger and having fewer comorbidities, patients with COVID-19 had a higher risk of severe illness [14]. Dupont et al [15] found worse outcomes in adults with asthma who had COVID-19 compared to those with asthma who had influenza. However, these studies did not focus on pediatric or asthma populations.

Most studies on COVID-19 in children with asthma indicate that coexisting asthma is linked to a poorer prognosis. For example, Robbins et al [16] reported that children with asthma seen in the emergency department for COVID-19 were more likely to have severe infections compared to those without asthma. Another study of 5510 patients with asthma, including 414 patients with COVID-19, found a positive correlation between COVID-19 severity and asthma symptoms [17]. Similarly, Lara et al [18] showed that asthma significantly increased the risk of hospitalization in children with COVID-19.

While many studies report that asthma worsens COVID-19 outcomes, some studies present conflicting results. Notably, Oliveira et al [19] observed that children with asthma hospitalized for COVID-19 had a significantly lower risk of death compared to children without asthma. Similarly, Dolby et al [20] reported in a large cohort study that individuals with mild or well-controlled asthma were not at increased risk of severe COVID-19 outcomes. In line with these findings, 1 study of 107 pediatric patients with COVID-19 found that asthma and allergic diseases were not risk factors for hospitalization [21]. Yilmaz et al [22] suggested that COVID-19 may have a milder course than influenza in children (not specifically those with asthma).

### Mechanisms

Both COVID-19 and influenza can exacerbate asthma symptoms, but our findings show that children with asthma admitted for COVID-19 had a higher risk of developing sepsis or shock compared to those admitted for influenza. COVID-19 is known to trigger a severe immune response or cytokine storm, leading to high systemic inflammation, rapid respiratory deterioration, and increased risk of sepsis and shock [23]. The SARS-CoV-2, compared to the influenza virus, can induce deeper immune dysregulation [24], which may amplify the underlying airway inflammation in asthma. Consistent with this heightened virulence, Brehm et al [14] observed a markedly higher mortality in immunocompromised adults with COVID-19 than with influenza (33% vs 11.6%; *P*=.01).

Conversely, we found that children with asthma admitted for influenza had a higher risk of secondary bacterial infections than those admitted for COVID-19. Influenza can directly damage the respiratory epithelium, impair mucociliary clearance, and allow bacterial pathogens to invade, leading to secondary infections such as bacterial pneumonia [25]. COVID-19, although capable of severe pneumonitis, generally causes less direct epithelial destruction than influenza [25,26]. Poorly controlled asthma may exacerbate these risks, as noted by Lansbury et al [26], who reported that bacterial coinfection rates in patients with COVID-19 were much lower than during influenza pandemics. This finding suggests that the routine use of antibiotics in patients with COVID-19 may not be necessary. Early in the pandemic, despite low rates of bacterial coinfections (<10%), antibiotics were widely prescribed to patients with COVID-19 (75%) [27]. Such practices might have reduced secondary bacterial infection rates, making patients with influenza more susceptible to secondary infections.

### Clinical Implications

Despite these differences in complications, we observed no significant difference in in-hospital mortality rates between the two groups. This could be due to effective clinical management or the resilience of the pediatric population. However, younger children (aged 1 to 5 years) with COVID-19 had a higher 90-day readmission rate than those with influenza, indicating a need for targeted follow-up care after discharge.

Studies have shown that COVID-19 vaccines are safe and effective for children, including those with asthma [6]. Ongoing research continues to support vaccination, particularly for those with uncontrolled asthma, who are at increased risk of severe disease and complications [6].

These findings can guide health care providers in optimizing care during respiratory viral outbreaks. The study underscores the need for different management strategies for children with asthma hospitalized with COVID-19 or influenza. Specifically, the higher risk of sepsis or shock in the COVID-19 cohort warrants proactive hemodynamic monitoring and early escalation of care. Vaccination and close follow-up, particularly for younger children with COVID-19, are crucial to prevent hospitalizations and manage complications.

### Strengths and Limitations

A strength of this study is its use of a large, nationally representative dataset, providing a comprehensive overview of hospitalizations across the United States. This broad scope enhances the generalizability of the findings and allows a detailed understanding of the differential impact of COVID-19 and influenza on children with asthma. In addition, the analysis included in-hospital data and readmission data, which are informative in assessing health care use for these patients.

However, this study still had several limitations. First, the retrospective, observational design limited causal inference and may have introduced selection bias. The absence of a healthy (noninfected) comparison group further constrained interpretation, and the single-year study period (2020) limited generalizability to later phases of the pandemic. Moreover, reliance on *ICD-10-CM* codes could underestimate milder comorbidities, and variations in coding accuracy may have affected our results.

Second, several key clinical parameters were unavailable in the NRD, such as vaccination history of patients, COVID-19 viral load, laboratory measures, influenza subtypes, detailed treatment regimens, asthma medication regimens (particularly inhaled corticosteroids), and asthma severity. These factors could significantly influence patient outcomes and limit the ability to fully understand the nuances of disease interactions in this population. Emerging evidence suggests that impaired fetal growth, rather than prematurity, influences childhood lung function; such perinatal data were similarly not captured [28].

Third, the COVID-19 and influenza groups differed markedly in age (mean 13.3 vs 6.8 years, respectively), raising the possibility of residual confounding. Although age-stratified analyses yielded trends but these results were not statistically significant (*P*=.12) consistent with the primary results, small subgroup sample sizes reduce statistical power—particularly for rare outcomes such as mortality.

Finally, clinical management of COVID-19 evolved rapidly during 2020, and pandemic-related changes in health care–seeking behavior may have biased our sample toward more severe cases. A nationwide Korean survey reported a transient decline in pediatric asthma prevalence from 2020 to 2021, potentially reflecting underdiagnosis or reduced health care use [29]; similar dynamics could have influenced the US data.

### Future Directions

Future work should couple national hospital data with electronic health records to add missing variables—vaccination status, asthma-control metrics, and viral subtype—across multiple post-2020 seasons. Including a group of noninfected children with asthma would clarify the absolute risk estimates, while extended follow-up may reveal longer-term monitoring.

### Conclusions

In conclusion, children with asthma in the United States hospitalized for COVID-19 have a higher risk of sepsis or shock compared to those admitted for influenza. Conversely, those admitted for influenza are at increased risk of secondary bacterial or fungal pneumonia. These findings underscore the need for targeted clinical management strategies for children with asthma, tailored to the specific risks posed by COVID-19 and influenza.

## Appendix

Multimedia Appendix 1 Supplementary Table 1 International Classification of Diseases, 10th Revision, Clinical Modification (ICD-10-CM) and Procedure Coding System (ICD-10-PCS) codes applied to identify diagnoses, complications, and procedures in the 2020 U.S. Nationwide Readmissions Database cohort of children with asthma hospitalized for COVID-19 or seasonal influenza.

## Abbreviations

aOR: adjusted odds ratio
HCUP: Health Care Cost and Utilization Project
ICD-10-CM: International Classification of Diseases, 10th Revision, Clinical Modification
ICD-10-PCS: International Classification of Diseases, 10th Revision, Procedure Coding System
IRB: institutional review board
NRD: Nationwide Readmissions Database
PROC SURVEYFREQ: survey frequency procedure
PROC SURVEYLOGISTIC: procedure for survey logistic regression analysis
PROC SURVEYREG: procedure for survey regression analysis

## Data Availability

All the data supporting underlying findings are included in the manuscript and its multimedia appendices.
