# Supplementary material for: Comparative Analysis of Outcomes of Influenza and COVID-19 Admissions Among Children With Asthma: A Nationwide Retrospective Cohort Study Using the US National Readmissions Database
Source: JMIR Med Inform. 2025 Sep 30;13:e73047. doi: 10.2196/73047 (PMC12521845; doi:10.2196/73047)
Supplement: Multimedia Appendix 1 [file medinform_v13i1e73047_app1.docx]

Supplementary Table 1. International Classification of Diseases, 10th Revision, Clinical Modification (ICD-10-CM) and Procedure Coding System (ICD-10-PCS) codes applied to identify diagnoses, complications, and procedures in the 2020 U.S. Nationwide Readmissions Database cohort of children with asthma hospitalized for COVID-19 or seasonal influenza.

|  | ICD 10 Code |
| --- | --- |
| Asthma | **CM**: J45 |
| COVID-19 | **CM**: U07.1, U00, U49, U50, U85, J12.82 |
| Influenza | **CM**: J10, J11, J09 |
| CVA | **CM**: M05, M06 |
| VTE | **CM**: I26.0, I26.9, I80.0-I80.3, I80.8, I80.9, I81, I82, O08.2, O22.3, O87.1, O88.2 |
| Sepsis | **CM**: R78.81, A41, R65.2, A42.7, A22.7, B37.7, A26.7, A28.2, A54.86, A32.7, A39.2, A20.7, A21.7, A48.3, A24.1 |
| Bacterial/Fungal pneumonia | **CM**: J13-J18 |
| Respiratory failure | **CM**: J95.2-J95.8, J96.00, J96.90, J80, J81.0 |
| Mechanical ventilation | **CM**: Z99.12  **PCS**: 5A1935Z, 5A1945Z, 5A1955Z |
| Acute kidney injury | **CM**: N17 |
| Shock | **CM**: R57, T81.1, T88.2, R65.21, A48.3 |
| Diabetes mellitus | **CM**: E10-E13 |
| Hypertension | **CM**: I10, O10.0, O10.9, I16, I67.4 |
| Obesity/overweight | **CM**: E66.x |
| Neurological disease | **CM**: G10.x-G13.x, G20.x-G22.x, G25.4, G25.5, G31.2, G31.8, G31.9, G32.x, G35.x-G37.x, G40.x, G41.x, G93.1, G93.4, R47.0, R56.x |
| Down Syndrome/chromosomal anomaly | **CM**: Q90.x |
| Metabolic disease | **CM**: E72, E74-E83 |
| Cystic fibrosis | **CM**: E84 |
| Amyloidosis | **CM**: E85 |
| Sickle cell disease | **CM**: D57.x |
| Congenital heart condition | **CM**: I09.9, I11.0, I13.0, I13.2, I25.5, I42.0, I42.5-I42.9, I43.x, I50.x, P29.0 |
| Congenital lung condition | **CM**: I27.8, I27.9, J40.x - J47.x, J60.x-J67.x, J68.4, J70.1, J70.3 |
| Autoimmune disease | **CM**: M32.x, M05.x, M06.x, G35.x, E03.9.x, K50.x, K51.x, L40.x |
| Disability | **CM**: F70.x-F79.x, R53.2.x, F84.x |

ICD, International Classification of Disease; CM, clinical modification; PCS, procedure coding system.
